# Development of an ecologically valid approach to assess moderate physical activity using accelerometry in community dwelling women of color: A cross-sectional study

**DOI:** 10.1186/1479-5868-8-21

**Published:** 2011-03-25

**Authors:** Charles S Layne, Scherezade K Mama, Jorge A Banda, Rebecca E Lee

**Affiliations:** 1 Texas Obesity Research Center, Department of Health and Human Performance, University of Houston, 3855 Holman Street, 104 Garrison Gym, Houston, TX 77204-6015, USA; 2 Department of Exercise Science, University of South Carolina, Columbia, SC, USA

## Abstract

**Background:**

Women of color report the lowest levels of physical activity and highest rates of overweight and obesity in the US. The purpose of this study was to develop an individualized, ecologically valid, field based method to assess physical activity over seven days for community dwelling women of color using accelerometers.

**Methods:**

Accelerometer-measured physical activity, Borg perceived exertion, demographics, blood pressure, heart rate, and anthropometric measures were collected from African American and Hispanic or Latina women (*N *= 209). A threshold for increased physical activity was determined for each participant by calculating the average count per minute (plus one standard deviation) for each participant collected during a self-selected pace that corresponded to a 'recreational' walk about their neighborhood. The threshold was then used to calculate the amount of time spent doing increased intensity physical activity during a typical week.

**Results:**

Women were middle-aged and obese (*M *BMI = 34.3 ± 9.3). The average individual activity counts per day ranged from 482-1368 in African American women and 470-1302 in Hispanic or Latina women. On average, African American women spent significantly more time doing what was labeled 'increased' physical activity than Hispanic and Latino women. However neither group approached recommended physical activity levels, as African American women, averaged 1.73% and Hispanic and Latino women averaged 0.83% of their day engaged in increased physical activity (*p *< 0.05).

**Conclusions:**

This study presents a simple field-based method for developing accelerometer thresholds that identify personalized thresholds of moderate intensity physical activity that can be used by in community-based settings. Findings highlight a need for physical activity programs whose starting points are based upon the individual's typical baseline physical activity level, which is likely to be well below the minimum recommended published guidelines.

## Background

It is well documented that physical activity helps control body weight, and is associated with a reduced risk of coronary heart disease, high blood pressure, diabetes, numerous cancers, and other health compromising conditions [1,2]. Although many studies are conducted to promote and understand the benefits of physical activity, measurement limitations plague these studies, owing to concerns about validity from participant reports and weaknesses in translation of laboratory measures to field settings, contributing to poor ecological validity. Ecological validity refers to the concept that for the results of a study to be generalizable to a larger population, the methods and the setting of the investigation must closely approximate the real-life situation under investigation [3]. Accurate methods of physical activity measurement are needed, particularly for those studies that provide ongoing surveillance of physical activity, identify correlates of physical activity adoption and maintenance, and assess the effectiveness of community interventions.

Many physical activity studies have used accelerometry, based on carefully controlled physical activity variables in laboratory settings to develop valid and reliable cut points directly related to metabolic equivalents (MET). It is difficult to replicate these carefully controlled laboratory studies in the field and community settings [4-6], suggesting significant problems associated with ecological validity, where conditions generated under exacting laboratory conditions do not generalize well to real life conditions [3]. Further, there has been little research on the population subgroups with the greatest need for effective interventions to increase physical activity, namely African American (AA) and Hispanic or Latina (HL) women, who consistently report the lowest levels of physical activity [6] and demonstrate significantly higher rates of overweight and obesity in the US [4]. These two groups of women may also have differences in physical activity levels as a result of living in different neighborhoods and experiencing different cultural influences, further pointing to the need to develop ecologically valid physical activity measures and associated guidelines.

Laboratory studies often focus on physical activities like running that are seldom done by community dwelling AA and HL women. There are few investigations of walking for physical activity in community settings that have used accelerometers to assess physical activity levels despite the fact walking is the most commonly recommended activity by health care providers Moreover, there are even fewer studies using accelerometry that provide information concerning the amount of physical activity done by AA and HL women in their usual environments [7,8]. There is a particular need to accurately identify useful physical activity measurement guidelines for community dwelling women of color.

Existing laboratory based cut point procedures and protocols and the resulting guidelines may provide little guidance for community based research and have limited generalizability for these particularly vulnerable populations. Laboratory based guidelines may not be ecologically valid, because the range of accelerometer-based activity counts that constitute moderate or greater intensity physical activity in laboratory samples may differ from those for community-based women of color. Inappropriate guidelines then in turn lead to recommendations for designing and implementing physical activity and weight loss and management programs for these populations that are not appropriate or useful. It has been reported that physical activity intervention program adherence rates are negatively correlated with the intensity of physical activity [9,10], making it all the more important to have appropriate guidelines for physical activity intervention programs. As funding agencies and scientists begin to recognize the lack of evidence required to accurately evaluate typical physical activity levels in community dwelling women of color, there is a vital need for ecologically valid and representative measurement strategies.

Research is needed to determine ecologically valid information regarding physical activity levels obtained from accelerometry; information that could be used to determine whether community based physical activity protocols during interventions should be modified to either increase or decrease the amount of physical activity prescribed for participants. The initial step in this process is to identify the accelerometry counts associated with naturally occurring physical activities, like walking, in typical community settings. Second, it is important to document the amount of physical activity actually done by community dwelling women, and then to identify personalized thresholds of physical activity directly related to the level of their self-selected walking pace that would represent a reasonable increase in physical activity for each individual participant. This information could be used to guide the development of ecologically valid physical activity guidelines in community dwelling women of color. Without more complete information concerning typical physical activity patterns in community dwelling women of color, it is difficult to determine the role that various factors have on physical activity, and whether the level of physical activity done by those in community-based intervention studies is adequate to demonstrate health benefits, such as weight loss and maintenance. The purpose of this study was to develop an ecologically valid, accelerometry-based methodology that is field-based, easy to implement, and provides a representative measure of usual physical activity. This methodology provides the ability to compute activity measurements obtained from community dwelling women in their daily living environments based on their current level physical activity.

## Method

### Participants

African American and Hispanic or Latina community-dwelling women (*N *= 209) from the cities of Houston and Austin, Texas were enrolled in Health Is Power, an ongoing study of physical activity and dietary habits, that has been previously described [8]. The Health Is Power project took place year-round between June 2006 and July 2008. As presented in Table 1, women who volunteered to participate in this study were typically middle aged and overweight or obese, but otherwise healthy. To qualify to participate in the study, women could not be doing more than 30 minutes of moderate to vigorous

**Table 1:** Participant Descriptive Statistics by Ethnicity

Variable	African American	Hispanic or Latina	Total
	*M *± *SD*	*M *± *SD*	*M *± *SD*
Age (years)	44.3 ± 10.8	46.6 ± 10.1	45.2 ± 10.5
BMI (kg/m2)	34.6 ± 9.7	33.9 ± 8.8	34.3 ± 9.3
% Body Fat	42.1 ± 9.2	41.9 ± 8.6	42.0 ± 8.9
Systolic BP (mmHg)	123.4 ± 21.4	123.0 ± 20.4	123.2 ± 21.0
Diastolic BP (mmHg)	77.7 ± 13.6	76.6 ± 13.0	77.3 ± 13.4
Heart Rate (BPM)	72.2 ± 12.8	73.2 ± 12.8	72.6 ± 12.8

This study was approved by the University's Committee for the Protection of Human Subjects, and participants provided their informed consent to participate. The investigators certified that all applicable institutional and governmental regulations concerning the ethical use of human research volunteers were followed during the investigation.

### Measures

Participants completed accelerometer derived measures of physical activity (see below), Borg Rating of Perceived Exertion (RPE), interviewer administered questionnaires of demographic information, and simple physical health assessments.

#### Physical Activity

Each participant was loaned an accelerometer for one week. This week began on the day of the orientation walk (see below) with the accelerometer returned following seven days of use. The participants were instructed to wear the accelerometer at all times, except when showering or sleeping, and to conduct themselves in a manner that represented a 'typical' week in terms of daily living physical activity. The accelerometers were returned after one week, and the data were downloaded, processed and subsequently analyzed.

*The Borg RPE scale *[11] was used in this study to evaluate the perceived level of intensity for each participant during the group walk. Participants were instructed on how to complete the Borg RPE scale prior to the group walk and completed a paper and pencil version of the scale after the group walk. The scale ranges from 6 to 20, with 6 corresponding to no exertion at all, 7.5 to extremely light, 9 to very light, 11 to light, 13 to somewhat hard, 15 to hard, 17 to very hard, 19 to extremely hard, and 20 to maximal exertion [11].

*Demographic information *on ethnicity, education, and family income were collected using the Multigroup Ethnic Identity Measure (MEIM) [12] and the Maternal and Infant Health Assessment (MIHA) survey [13]. Using an interviewer administered survey, participants identified their ethnicity, highest level of education, total family pre-tax income for the most recent tax year, and the number of people living on this income.

#### Physical health assessment

Participant height, weight, resting pulse rate, resting blood pressure, and percent body fat were measured by trained research staff using standardized protocols [14]. Height was measured to the nearest centimeter using a portable stadiometer. Participants removed their shoes, stood with their back facing the stadiometer, stood as straight as possible, and looked forward during height measurement. Body weight and percent body fat were measured using a Tanita TBF-310 body composition analyzer. Participants were asked to remove their shoes, socks, and excess clothing. Resting pulse rate and resting blood pressure were measured after research participants sat comfortably for at least 10 minutes. Pulse rate was measured twice manually for one minute at the radial artery of the left wrist. Resting blood pressure was measured twice manually on the left arm with a hand aneroid sphygmomanometer and stethoscope. All physical health assessment measures were collected twice, and the average of the two values was used in analysis.

### Procedure

#### Group Orientation Walk

A 12 minute group orientation walk was completed to obtain a measure of accelerometer counts representing a pace consistent with the pace the participants would employ during a 'recreational' walk around their neighborhood or community park. Each participant wore an ActiGraph GT1 M accelerometer at her hip during the group walk and completed the Borg RPE scale [11] after the walk. Each participant completed one group walk; ten group walks were conducted over the course of the study, with 16 to 27 participants participating in each walk. Group walks were monitored by a senior research staff member, who instructed the participants to walk at their own comfortable pace consistent with a leisurely, recreational walk. The group walk occurred in the early evening around a designated path in a large, outdoor parking lot where there was no automobile traffic. This allowed each participant to self-select a pace that was comfortable for her without losing sight of the group or assembly area. The purpose of this walk was to provide a measure of the pace, and associated activity counts, the women would typically adopt were they to take a leisurely walk on their own.

### Data Processing and Statistical Analysis

Accelerometers were programmed and initialized to collect activity counts at a one minute epoch setting. Using the ActiLife software [15] that accompanied the accelerometers, .DAT files were downloaded from the accelerometer and used to create an Excel file that contained activity counts. The first step in data processing was to identify the number of one minute epochs per day in which activity counts were recorded. The files were then evaluated to determine whether the participants had worn the accelerometer at least eight hours per day. Given there are 1440 minutes in a 24 hour day, the participants had to demonstrate accelerometer counts in at least 480 epochs. If so, accelerometer activity from that day was included in the subsequent analysis [16]. If the accelerometer was not worn for eight hours on a given day, then available data from that day were not included in analysis. The next step was to determine whether the participant wore the accelerometer for at least four days during the week that the device was in her possession. If the participant met the four day minimum requirement, then the data from the four or more days when she wore the accelerometer were subjected to further analysis [16]. For each participant, means and standard deviations of the total activity counts per day based on the number of days the accelerometer was worn were computed. To summarize, the acceptance criteria for accelerometer data to be included in the data set included the accelerometer being worn at least eight hours per day for four or more days within a seven day period.

To determine whether an individual participated in what we considered moderate physical activity for her, several data processing steps were taken. For each participant, the activity counts during the group orientation walk were averaged over the walk. We considered this mean to represent a low level of physical activity. The standard deviation of the accelerometer counts of the 12 minute walk was then calculated and the value equal to the average plus one standard deviation was obtained. This value was used to represent the threshold for what was considered 'increased' physical activity for each individual participant. The accepted data, based on the procedures above, were then tabulated to determine how many minutes per day were spent in increased physical activity. This value was then converted to a percentage of the day spent in increased physical activity based upon a 24 hour day and descriptive statistics (i.e. mean and standard deviations) for each day, for each participant, were computed. Grand means for each day of the data collection period were then calculated. The data were then grouped by ethnicity and Student t-tests were used to assess whether there were significant differences in physical activity levels between AA and HL women. An alpha level of *p *< 0.05 was adopted to signify significant statistical differences.

## Results

**Figure 1:**
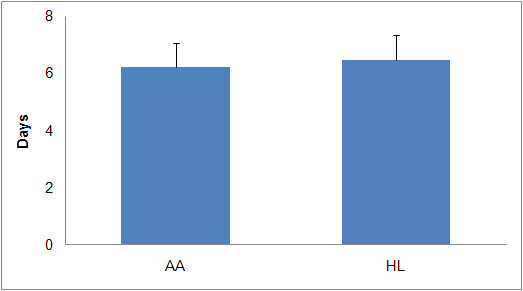
**Mean (+ 1SD) Number of Days Accelerometers were Worn in One Week**.

**Figure 2:**
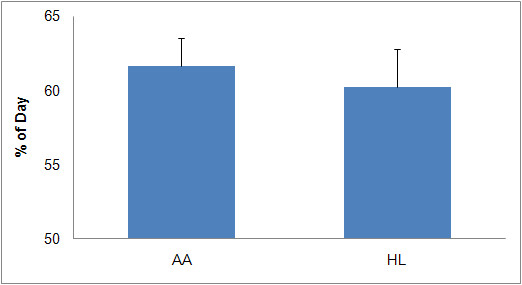
**Mean (+ 1SD) Percentage of Day Accelerometers were Worn in One Week**.

Participant descriptive statistics by ethnicity are reported in Table 1. Table 1 shows that the participants were typically middle aged and overweight or obese. Table 1 also shows that the sample's average pulse rate and diastolic blood pressure fell within healthy ranges (60-90 beats per minute and less than or equal to 80 mmHg, respectively), and the average systolic blood pressure fell within the pre-hypertension category (121-129 mmHg). Of the 209 women who wore an accelerometer following the orientation walk, 188 wore it for at least eight hours for four or more days a week. Thus, of the initial participant sample, 90.4% provided data that were subjected to additional analyses. The data set contained 117 AA women and 71 HL women who met the inclusion criteria. Figure 1 displays the mean number of days the accelerometers were worn during the assessment week, separated by ethnicity. Figure 2 displays the mean percentage of each day the participants wore the accelerometer, grouped by ethnicity.

**Figure 3:**
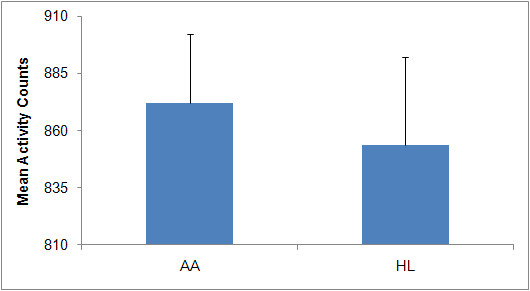
**Mean (+ 1SD) Number of Activity Counts Per Week**.

The percentage of the day the accelerometer was worn ranged from 33.6 to 91.7% in HL women and from 33.5 to 96.2% in AA women. Neither the mean percent of day nor the mean number of days the accelerometer was worn differed by ethnicity. Figure 3 displays the mean number of activity counts for AA and HL women across one week of accelerometer usage. The range of individual participant activity counts for AA women was from 482 to 1368, while the corresponding range for HL women was from 470 to 1302. There was no significant difference in the average activity counts between the two groups.

During the orientation walk, AA women showed significantly fewer activity counts than did the HL women. AA women averaged 1567.6 counts (SD = 243.9), while HL women averaged 1991.2 counts (SD = 382.1) (*p *< 0.05), indicating the HL women walked faster than the AA women. Borg ratings following the orientation walk average 9.2 (*SD *= 3.8) for AA women and 10.4 (*SD *= 2.8) for HL women. The values for both groups correspond to the perception of very light exertion. The data revealed no differences across different orientation walk cohorts that could be attributed to possible seasonal effects. To determine whether the participants engaged in what we labeled 'increased' physical activity the number of one minute increments spent in physical activity that corresponded to the mean plus one standard deviation or more above the mean activity count obtained during the orientation walk, was tabulated for each participant. The average thresholds for increased physical activity for the AA women and the HL women were 1811.5 and 2373.3 counts per minute, respectively. For the AA women the thresholds values ranged from 676.2 - 4018.5 (median 1679.4). For HL women the threshold values ranged from 1255.5 to 5474.7 (median 2320.7). Matthews [17] reported a median moderate exercise cut point of approximately 2100 that was calculated for studies that developed cut point equations for walking and running derived from ActiGraph accelerometer counts. Guinhouya et al [18] suggested a moderate exercise cut point of no less than 3000 counts per minute when assessing children. Seventy four percent of AA women did not reach an activity count value of 2100 per minute while 63% of HL women exceeded that value during the orientation walk.

**Figure 4:**
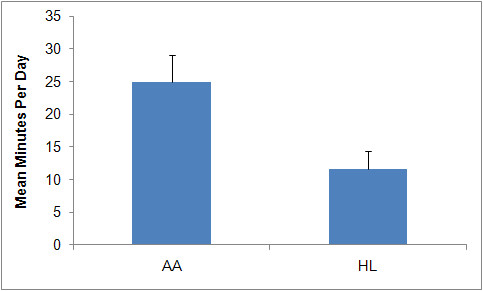
**Mean (+1SD) Number of Minutes Per Week Engaged in Daily Increased Physical Activity**.

Figure 4 displays the mean number of minutes per day spent in increased physical activity during the assessment week by ethnic group. African American women spent a significantly greater amount of time engaged in increased physical compared to HL women. On average, AA women spent 1.73% (*SD *= 0.27) of their day engaged in increased physical activity, while HL women only spent 0.83% (*SD *= 0.19) of their day engaged in increased physical activity (*p *< 0.05). Most (54.7% of AA and 90.1% of HL) participants averaged fewer than 20 minutes of increased physical activity per day, indicating these women were typically inactive based upon our methodology.

## Discussion

Individualized, tailored measures of physical activity are gaining in popularity, as they are more accurate in estimating the amount of time spent in different intensity levels than a group based measure [19,20]. Unlike previous studies conducted in laboratory settings with protocols unrealizable in community settings, the method presented herein is (1) relatively simple to conduct, (2) useful for those with limited laboratory techniques training, and (3) suitable for community based research settings as a simple but ecologically valid way to measure the amount of time spent in different physical activity intensities.

Previous studies required the use of expensive, indirect calorimetry equipment, the use of a treadmill, and required participants to spend a large amount of time in a laboratory. The purpose of this study was to develop an ecologically valid approach to measure physical activity levels in community dwelling, women of color and document the amount of physical activity done during a typical week. Such an approach, and the data derived from it, could be used by other investigators involved in community based interventions, to design personalized physical activity programs. The methodology presented here is novel in that the documented amount of increased physical activity is calculated relative to each participant's self-selected and self-identified recreational walking pace. The ecological validity of the measure provides assurances to those implementing community based physical activity intervention programs that each participant is doing a level of physical activity that exceeds their typical physical activity level but is unlikely to be so taxing as to discourage the participants and lead to drop out. Other than the cost associated with accelerometers and weight and height measuring devices, no other equipment was needed for the described method. This method can also be completed in a short amount of time and in a community based setting, placing little burden on research or intervention program participants.

The decision to label the mean plus one SD of the activity counts obtained during the orientation walk as 'increased' physical activity is intended to illustrate how the methodology can be used to personalize the development of individualized physical activity thresholds for ecologically accurate measurement and intervention milestones. Alternatives to using standard deviations to establish intervention goals could include the selection of an absolute number of activity counts or a simple percentage increase beyond a given baseline level. Regardless, a baseline level of physical activity should be tied to a self-selected walking pace that corresponds to a Borg scale exertion level of 'very light'. Given that our participants spent very little time in what we defined as increased physical activity, establishing mean baseline values upon data obtained during more vigorous activities than our orientation walk pace and then establishing physical activity goals much above that baseline are unlikely to be met by the populations included in this study.

Although it would have been interesting to incorporate a broader range of physical activities into the development of our measure, we felt comfortable anchoring our measure to that of a 'recreational' walking pace, since walking is the most commonly reported mode of physical activity in the US and is typically cited by public health recommendations as appropriate for the general population [21]. Despite using our relatively low threshold for increased physical activity tied to individual participant's comfortable walking pace, we identified low levels of physical activity done by our sample. Although our inclusion criteria required that the participants exercise fewer than 90 minutes per week, our data confirmed previously reported data [22] on the dearth of physical activity done by these segments of the US population. Identifying the mean activity counts associated with the orientation walk plus one standard deviation resulted in a relatively low threshold of physical activity particularly for the AA women. This fact points to the need to develop physical activity programs whose starting points are based upon the individual's typical baseline physical activity level, which are likely to be below the minimum recommended published guidelines. Prescribing physical activity levels well beyond an individual's typical physical activity level presents the possibility of a host of acute negative physical health consequences, loss of motivation, and failure to adopt and maintain physical activity. The negative relationship between the intensity of physical activity and intervention program adherence suggests that it is important to initially identify personalized levels of physical activity that the participants are comfortable performing so as to increase the likelihood of adherence during the intervention and beyond [9,10].

Although not the focus of this paper, we did identify that, on average, AA women spent significantly more time in increased physical activity compared to HL women. Given that the AA women walked at a slower pace during the orientation walk (i.e. fewer counts per minutes) than the HL women, AA women had, on average, lower thresholds for identification of increased physical activity, based upon our methodology. Thus, AA women would need to do lower levels of physical activity during the data collection period to be identified as involving in increased physical activity. Conversely, HL women, on average, walked at an orientation walk pace that was consistent with 'moderate' exercise meaning they required a greater level of physical activity to be labeled as engaged in increased activity [17]. As noted, they reached this threshold of physical activity during less than one percent of the data collection period. There are a variety of potential reasons for these related findings, including differences between AA and HL women regarding family and socioeconomic status differences, cultural norms associated with physical activity, nutrition and other health habits, personal motivations, and built environment issues. These findings reinforce the contention that ecologically valid measures of physical activity are of critical importance. Regardless of the reasons for the identified differences, it remains important to focus on the fact that neither of our ethnic groups approached the recommended weekly amount of physical activity that would contribute to a healthy lifestyle, let alone weight loss or weight loss maintenance [23]. Although our inclusion criterion of fewer than 90 minutes per week of exercise ensured our sample was 'sedentary', our data indicate than none of the participants even approached the 90 minutes threshold.

## Conclusions

In summary, we have described a novel, field-based and ecologically valid methodology to measure total physical activity over the span of seven days for use with community dwelling women of color. By directly relating each individual participant's threshold for increased physical activity to her own self-selected walking pace (mean plus one SD), as determined from accelerometer counts obtained during a 'recreational' walk, this easily calculated measure can be used by physical activity specialists to prescribe a healthy level of physical activity for each individual engaged in a intervention program.

## Competing interests

The authors declare that they have no competing interests.

## Authors' contributions

CSL led the writing and analyses and assisted with the study. SKM assisted with the study, analyses, writing and formatting of the manuscript. JAB assisted with the downloading and processing of accelerometer data and writing. REL led the study and assisted with writing. All authors read and approved the final manuscript.
